# Supplementary material for: Diversity and Antibiotic Resistance of Triticale Seed-Borne Bacteria on the Tibetan Plateau
Source: Microorganisms. 2024 Mar 25;12(4):650. doi: 10.3390/microorganisms12040650 (PMC11052201; doi:10.3390/microorganisms12040650)
Supplement: Supplementary file 1 [file microorganisms-12-00650-s001.zip › microorganisms-2899260-supplementary.pdf]

Supplementary data

Table S1: Growth distribution of 40 μg/mL antibiotic plate strains.

Strain	Antibiotic								
	TET	AMP	CPFX	SUD	RFD	AMI	OTC	KAN	EM
G1-1	-	-	-	-	-	-	-	-	-
G1-3	-	-	-	-	-	-	-	-	-
G1-4	-	-	-	-	-	-	-	-	+
G1-5	-	+	-	-	-	+	-	-	-
G2-16	-	+	-	+	+	+	-	+	+
G2-17	-	+	-	+	-	-	-	-	+
G2-4	-	-	-	-	-	+	-	-	-
G3-1	-	-	-	-	-	-	-	-	-
G3-3	-	-	-	-	-	-	-	-	-
G3-4	-	-	-	-	-	-	-	-	-
G3-8	+	-	-	-	-	-	+	-	-
G2-10	-	+	-	-	-	-	-	-	-
G4-11	-	-	-	+	-	-	-	-	-
G4-7	-	+	-	+	-	+	-	-	+
G4-8	-	-	-	-	-	-	-	-	-
G4-9	-	+	-	-	-	-	-	-	-
G7-4	-	-	-	-	-	-	-	-	-
G7-5	-	-	-	-	-	-	-	-	-
G7-7	-	-	-	-	-	-	-	-	-
G7-8	-	-	-	-	-	-	-	-	+
S1-4	-	-	-	-	-	-	-	-	-
S1-5	-	+	-	+	-	-	-	-	-
S1-7	-	+	-	-	-	-	-	-	-
XG1-3	-	+	-	-	-	-	-	-	-
XG1-8	-	-	-	-	-	-	-	-	-
XG3-10	-	-	-	-	-	-	-	-	-
XG3-8	-	+	+	+	+	+	-	-	+
XG4-8	-	+	-	+	-	-	-	-	-
XG7-3	-	-	-	-	-	-	-	-	-
XG7-6	-	-	-	-	-	-	-	-	-
XZ2-1	-	-	-	-	-	-	-	-	-
Z2-19	-	-	-	-	-	-	-	-	-
Z2-6	-	-	-	-	-	-	-	-	-
Z2-5	-	-	-	+	-	-	-	-	-
ZS-1	-	+	-	+	-	-	-	-	-
ZS-2	-	-	-	-	-	-	-	-	-
ZS-5	-	+	-	-	-	-	-	-	-

' + ' indicates that the strain can be grown on antibiotic plates at that concentration, and vice versa is indicated by ' - '.

Table S2: The diameter of the inhibition zone of different concentrations of antibiotics on bacteria

Strain	Antibiotic				Inhibition rate (*I*)	
	Name	Concentration (μg/mL)				
		CK	40	640	40	640
G1-4	EM	8	8	9.3	-	16.30%
G1-5	AMP	20.94±0.229	22.02±0.229	24.70±0.383	5.20%	18.00%
G1-5	AMI	8	22.68±1.998	*	183.50%	*
G2-17	AMP	10.36±0.378	11.10±0.348	11.6±0.591	7.10%	12.00%
G2-17	EM	8	8.28±0.086	9.64±0.330	3.50%	20.50%
G2-17	KAN	8	9.38±0.166	12.60±0.100	17.30%	57.50%
G2-17	SUD	8	8	8	-	-
G2-4	AMI	8	8	10.66±0.299	0.00%	33.30%
G3-8	OTC	8.96±0.178	9.46±0.093	10.14±0.144	5.60%	13.20%
G3-8	TET	8	9.70±0.200	13.10±0.400	21.30%	63.80%
G4-10	AMP	12.28±0.166	13.12±0.15	15.26±0.308	6.80%	24.30%
G4-11	SUD	8	8	11.42±0.188	-	42.80%
G4-7	AMP	15.84±0.199	16.80±0.200	18.12±0.235	6.10%	14.40%
G4-7	SUD	8	8	8	-	-
G4-7	AMI	8	19.38±0.334	*	142.30%	*
G4-7	EM	8	8	8	-	-
G4-9	AMP	13.72±0.304	14.56±0.256	15.14±0.339	6.10%	10.30%
G7-8	EM	8	8	8	-	-
S1-5	SUD	8	8	8	-	-
S1-7	AMP	13.78±0.273	15.36±0.304	18.18±0.213	11.50%	31.90%
XG1-3	AMP	12.26±0.163	12.86±0.144	14.82±0.235	4.90%	20.90%
XG3-8	CPFX	10.30±0.224	10.92±0.287	18.72±0.185	6.00%	81.70%
XG3-8	AMP	10.28±0.208	11.34±0.268	12.52±0.267	10.30%	21.80%
XG3-8	AMI	8	15.14±0.121	*	89.30%	*
XG3-8	RFD	8	12.20±0.122	20.30±0.436	52.50%	153.80%
XG3-8	EM	8	8.5	13.10±0.400	6.30%	63.80%
XG3-8	SUD	8	9.12±0.188	9.80±0.292	14.00%	22.50%
XG4-8	SUD	8	8	11.46±0.150	0.00%	43.30%
XG4-8	AMP	8	8	11.24±0.196	0.00%	40.50%
Z2-5	SUD	8	8	8	-	-
ZS-1	AMP	12.06±0.150	13.00±0.221	14.22±0.325	7.80%	17.90%
ZS-1	SUD	8	8	10.58±0.282	0.00%	32.30%
ZS-5	AMP	13.64±0.214	14.64±0.204	15.38±0.421	7.30%	12.80%

' *' indicates that the inhibition rate of the antibiotic at a concentration of 40 μg/mL was greater than the IC_{50} of the strain; thus, the antibiotic was no longer effective at a concentration of 640 μg/mL. ' - ' indicates that the antibiotic does not produce a significant inhibition zone on the strain at the corresponding concentration, and its inhibition rate is approximately ' 0'.
